# Monomethyl lithospermate alleviates ischemic stroke injury in middle cerebral artery occlusion mice *in vivo* and protects oxygen glucose deprivation/reoxygenation induced SHSY-5Y cells *in vitro via* activation of PI3K/Akt signaling

**DOI:** 10.3389/fphar.2022.1024439

**Published:** 2022-10-13

**Authors:** Fang Yang, Ze-Ran Chen, Xu-Hong Yang, Yue Xu, Ning-Jing Ran, Mei-Jun Liu, Shuo-Guo Jin, Hua-Nan Jia, Yang Zhang

**Affiliations:** ^1^ Hospital of Chengdu University of Traditional Chinese Medicine, Chengdu, China; ^2^ School of Medical and Life Sciences, Chengdu University of Traditional Chinese Medicine, Chengdu, China

**Keywords:** stroke, ischemic stroke, ischemia-reperfusion, neural damage, PI3K/Akt pathway, OGR/R

## Abstract

Stroke is a fatal neurological disease, which seriously threatens human health and life. Ischemic stroke (IS) is the most common type of stroke in clinic. Its pathogenesis is very complex, mainly caused by nerve damage caused by brain blood supply disorder. Previous studies have confirmed that natural products play important roles in improving neurological disorders. Furthermore, our previous results also suggested that *Shenxiong Tongmai* granule, a clinically used herbal medicines’ prescription, has a good ameliorating effect on IS. In the present study, we found that Monomethyl lithospermate (MOL), a constituent of *Shenxiong Tongmai* granule, significantly improved the neurological damage in middle cerebral artery occlusion (MCAO) rats. MOL can significantly improve the neurological deficit score of MCAO rats, and improve the damage of hippocampal neurons caused by ischemia-reperfusion (IR). At the same time, we also found that MOL could reduce the level of oxidative stress in the brain tissues of MCAO rats. Furthermore, the oxygen and glucose deprivation/Reoxygenation (OGD/R)-induced SHSY-5Y cell model was established *in vitro* to investigate the pharmacological activity and molecular mechanisms of MOL in improving the nerve injury of IS rats. The results showed that MOL could increase the cell viability of SHSY-5Y cells, inhibit the mitochondrial membrane potential (MMOP) collapse and suppress apoptosis. In addition, MOL also ameliorated the elevated oxidative stress level caused by OGR/R treatment in SHSY-5Y cells. Further mechanistic studies showed that MOL could activate the PI3K/AKT pathway *via* promoting the phosphorylation of PI3K and AKT in MCAO rats and OGR/R-induced SHSY-5Y cells, which could be partially blocked by addition of PI3K/AKT pathway inhibitor of LY294002. Taken together, our current study suggested that MOL exerts a protective effect against neural damage caused by IS *in vivo* and *in vitro* by activating the PI3K/AKT pathway.

## 1 Introduction

Stroke is a serious clinically fatal disease with high morbidity, mortality, disability rate and other characteristics, which seriously threatens human health and life (Feigin et al., 2014). Stroke is mainly divided into hemorrhagic stroke (HS) and ischemic stroke (IS), and IS is the most common type of stroke in clinic, accounting for about 60%–80% of all strokes (GBD 2019). The pathogenesis of IS is very complex, mainly due to the disturbance of blood supply to the brain. Factors such as ischemia and hypoxia can cause ischemic necrosis of local brain tissue or encephalomalacia (Heiss, 2014). The main clinical treatment measures for IS is thrombolysis, thrombectomy, anti-platelet aggregation and nerve nutrition, *etc*., to restore the cerebral blood flow in the ischemic area as soon as possible and promote the recovery of neurological function (Catanese et al., 2017). However, the use of these therapies is limited by tight time windows and the risk of bleeding. In addition, reperfusion after cerebral IS can cause a large numbers of apoptosis of nerve cells, which is an important reason for the severe irreversible damage of brain tissue caused by IS. At present, increasing studies have confirmed that PI3K/Akt pathway plays an important role in regulating cell life activities, participating in a variety of physiological activities such as cell growth, differentiation, survival, apoptosis and so on (Cheng et al., 2017). In addition, studies have also shown that PI3K/Akt signaling pathway is also involved in a variety of programmed cell death in brain tissue injury, including apoptosis and autophagy (Tu et al., 2016; Lv et al., 2019; Samakov et al., 2019; He et al., 2021). Liu et al. demonstrated that PI3K/Akt signaling pathway can play an important role in the course of IS by regulating oxidative stress and inflammatory response (Liu et al., 2019).

**FIGURE 1:**
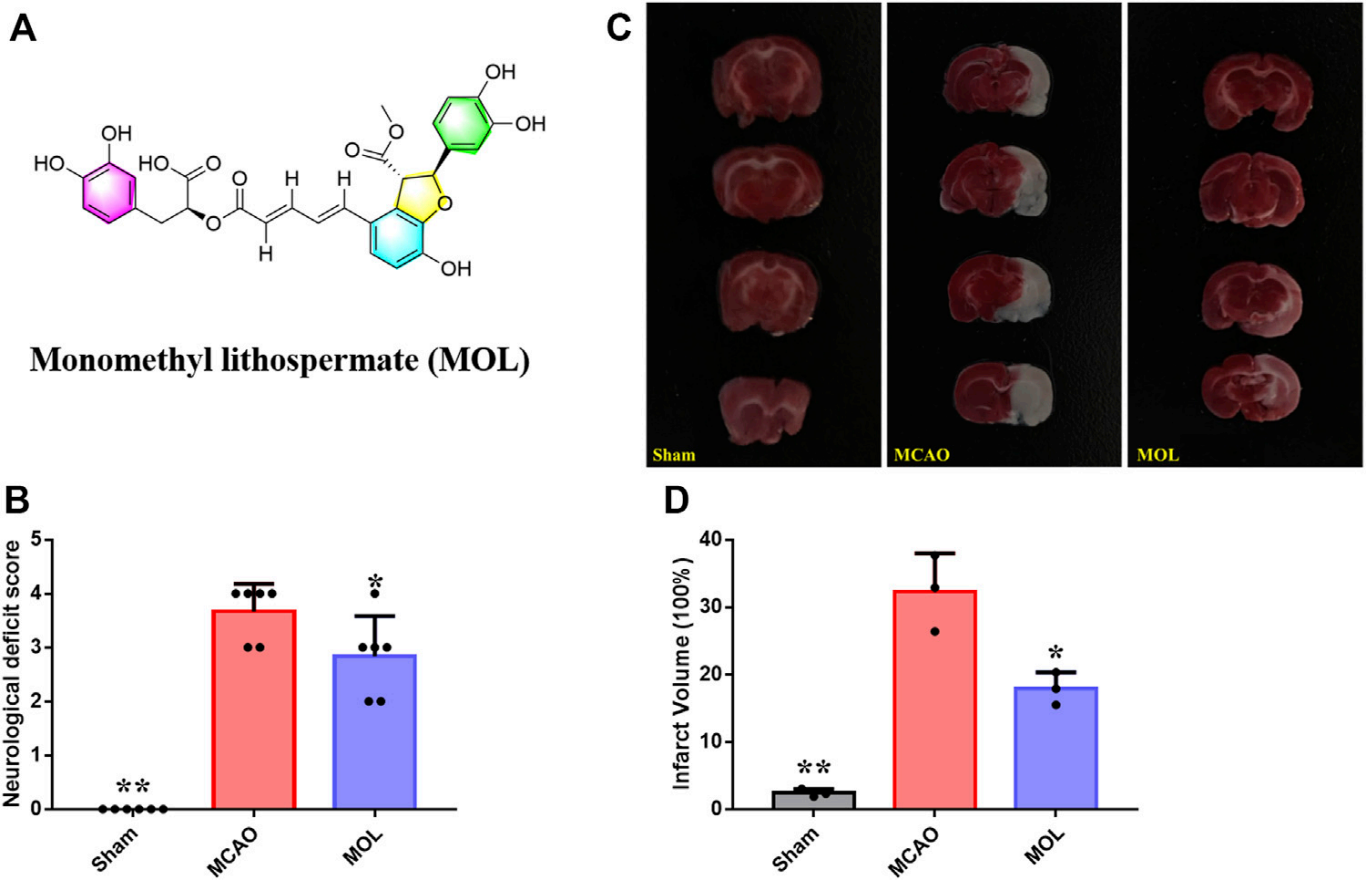
MOL can improve the neurological function of MCAO rats and reduce the area of cerebral infarction. **(A)** Chemical structure of MOL; **(B)** Improve the neurological deficit score of MCAO rats; **(C)** Effect of MOL on infarct size in MCAO rats; **(D)** Statistics of cerebral infarct size in MCAO rats. Data expressed as Mean ± SD, **p < 0.01, *p < 0.05 vs. MCAO group.

In recent years, drugs derived from natural plants have attracted the attention of many pharmaceutical researchers because of their good pharmacological activity and high safety (Zhang Q. et al., 2021). In our previous study, we also found that *Shenxiong Tongmai* granule, a clinically derived herbal medicines’ prescription, had obvious protective effect on nerve cell injury in cerebral IS rats (Ma et al., 2012; Wang and Chen., 2016). In this study, we found that the monomethyl lithospermate (MOL, Figure 1A), a component of *Shenxiong Tongmai* granule, could significantly improve nerve injury and reduce nerve cell apoptosis in middle cerebral artery occlusion (MCAO) rats. Further mechanistic studies have found that the protective effect of MOL on nerve cells is related to the activation of the PI3K/Akt pathway, and the addition of inhibitor LY294002 can partially reduce the neuroprotective effect of MOL.

## 2 Materials and methods

### 2.1 Chemicals and regents

MOL was purchased from the Chengdu HERBSource Biotech. Ltd. Co. (http://www.cdbcty.com/, Chengdu, China). with a purity over 95%. Tunel staining kits were purchased from Elabscience Biotechnology Co., Ltd. (Wuhan, China). The SOD, MDA, GSH and CAT detection kits were obtained from Suzhou Michy Biomedical Technology Co., Ltd. (Suzhou, China). CCK8 kit was purchased in Shanghai Dongren Chemical Technology Co., Ltd. (Shanghai, China). AO/EB Staining Kit was purchased in Shanghai Zeye Biotechnology Co., Ltd. (Shanghai, China). The JC-1 probe, DHE probe and apoptosis kit were purchased at Shanghai Baisai Biotechnology Co., LTD. (Shanghai, China). Primary antibodies against p-PI3K, PI3K, p-AKT and AKT were purchased from Wuhan ABclonal Technology Co., Ltd. (Wuhan, China). Goat polyclonal secondary antibody against rabbit/mouse for WB and IF were supplied by Abcam (Cambridge, MA, United States). LY2940029 was purchased from MedChemExpress (Shanghai, China).

### 2.2 Animal grouping

A total of 40 male Sprague-Dawley (SD) rats (220–260 g) were purchased from the Chengdu *Dasuo* experimental animal center and kept in a stable environment of 22°C–25°C and 45%–65% humidity. The environment had a circadian light-dark alter country of 12:12, and animals were adaptively fed for 5 days before the start of the experiment, with free access to food and water. Animal studies were approved by the Animal Care and Ethics Committee of Chengdu University of Traditional Chinese Medicine (No. 2020–07–4). The rats were randomly divided into three groups, including control group (sham operation group), treatment group (MOL) and model group. The treatment group was given MOL intervention in advance, 72.4 μM/kg MOL per day by gavage for 14 days. Rats in the sham operation group and the model group were gave with the corresponding volume of CMC-Na solution every day. On day 15, a slight improvement on our previous method was made to prepare the MCAO model (Ma et al., 2012; Wang and Chen., 2016). In brief, the rats were anesthetized with sodium pentobarbital, and the rats in the control group were sham-operated. The right common carotid artery structure of the rats was exposed to the bifurcation, and the skin was sutured. In the remaining rats, the distal end of the external carotid artery was ligated using silk thread, while another silk thread was threaded through the external carotid artery and a slipper knot was made close to the common carotid artery bifurcation. The common carotid artery was then clipped using an arterial clip, a small opening was cut in the external carotid artery 3 mm from the bifurcation of the common carotid artery, and a nylon thread with a treated tip was inserted into the small opening, into the internal carotid artery, and inward into the middle cerebral artery. The insertion depth of the nylon thread was approximately 16 ± 1 mm from the bifurcation. At 60min after ischemia, the thread plug was removed, the proximal end of the external artery was ligated with silk thread, and the neck wound was sutured. The wound was sterilized with iodine, and the rats were placed on a heating pad and placed in a constant temperature incubator after awakening.

### 2.3 Evaluation of neurological deficit of rats

At 24 h after operation, the behavioral test of rats was performed according to the modified Zea Longa standard to preliminarily judge and score the neurological deficit (Bederson et al., 1986). 0, No nerve damage; 1, Inability to extend the contralateral forelimb; 2, Contralateral forelimb flexion; 3, Turn slightly to the contralateral side; 4, There was no autonomic activity Heiss, 2014 with disturbance of consciousness.

### 2.4 Measurement of infarct size in rats (TTC staining)

At 24 h after surgery, the rats were deeply anesthetized with sodium pentobarbital, the skull was opened, the whole brain was removed, and the brain was rinsed using pre-cooled 0.9% sodium chloride solution. The slices were frozen at 20°C for 20 min, and then coronal slices of brain tissue were isometric, with a thickness of about 2 mm. The slices were placed in 2%TTC staining solution in a temperature chamber at 37°C for 15 min, and the slices were turned every 5 min to ensure uniform staining of brain tissue. The brain slices were fixed in 4% paraformaldehyde solution for 24 h, where the red part was normal tissue and the white part was infarcted tissue. The images were analyzed by ImageJ software. In order to exclude the influence of brain edema on the determination of cerebral infarction volume, the corrected calculation formula was used to calculate the percentage of cerebral infarction volume:

Percentage of cerebral infarct volume = (Volume of normal brain tissue contralateral to ischemia—Volume of normal brain tissue on the ischemic side)/Volume of normal brain tissue contralateral to ischemia x 100%

### 2.5 HE staining and nissl staining were performed

After anesthetizing mice, whole brains were removed and fixed in 4% paraformaldehyde. Subsequently, paraffin sections were prepared. HE staining and Nissl staining were performed to observe the pathological changes of rat brain tissue and neuronal damage.

### 2.6 TUNEL staining

After brain tissue sectioning, sections were stained according to the instructions of the Tunel staining kit. At the end of staining, the nuclei were labeled with DAPI, and the Tunel positive staining cells in the infarct area were observed by laser confocal microscopy to evaluate the level of neuronal apoptosis.

### 2.7 Cell culture and treatment

SHSY-5Y cells were purchased in Beijing Beina-Chuanglian biotechnology research institute, and cultured in DMEM medium supplemented with 10% FBS. Before starting the formal experiments, we examined the effect of MOL (1–100 μM) intervention on cell viability of SHSY-5Y cells after 12 h using the CCK8 kit. In a slight modification based on the method of Lu and Meng et al., oxygen and glucose deprivation/Reoxygenation (OGD/R) was performed using SHSY-5Y cells to simulate brain injury of I/R (Lu et al., 2011; Wang et al., 2014). Briefly, SHSY-5Y cells were placed into an anaerobic chamber and cultured in sugar-free Locke’s medium for 4 h. Then, the cells were transferred to normoxic conditions and the medium was replaced with normal medium, and the cells were re-oxidized for 12 h. In MOL treatment group, ODG/R-treated SHSY-5Y cells were treated with MOL (5, 10, 20 μM) for 12 h. In the inhibitor treatment group, MOL and 10 μM LY294002 were used to treat the cells for 12 h. At the end of treatment, CCK8 kit was used to detect the survival rate of cells in each group.

### 2.8 AO—EB staining

SHSY-5Y cells in the logarithmic growth phase were seeded into confocal laser dishes, and ODG/R and drug treatment were performed after the cells were completely adherent. At the end of the experiment, AO-EB staining working solution was added to the small dish and incubated for 5 min in the dark. After washing the cells three times with PBS, the fluorescence changes in the cells were observed by laser confocal microscopy and images were collected.

### 2.9 Cell mitochondrial membrane potential detection

The JC-1 probe was used to detect changes in MMOP in cells. At the end of the drug intervention, the medium was discarded, JC-1 staining solution was added and the cells were incubated in the dark for 30 min, and then the cells were washed three times with PBS. Laser confocal microscopy was used to detect the fluorescence changes in cells and acquire images.

### 2.10 Detection of apoptosis

At the end of drug intervention, the cells were collected and washed three times with PBS, then 5 μL Annexin V and 5 μL PI reagent were added and incubated in the dark for 30 min. The apoptosis rate of cells in each group was detected by flow cytometry.

### 2.11 ROS detection

ROS levels in cells were measured using DHE probes. At the end of the drug intervention, DHE probe was added to the laser confocal small dish and incubated for 30 min away from light. At the end of incubation, the cells were harvested and changes in ROS levels in the cells were determined using flow cytometry.

### 2.12 Oxidative stress level detection

The ischemic part of the brain tissue was homogenized at low temperature and centrifuged, and the supernatant was used to measure the protein concentration. For *in vitro* cell experiments, at the end of the drug intervention, the cells were harvested, broken and centrifuged, and the supernatant was taken for protein concentration determination. Finally, the levels of CAT, MDA, GSH and SOD in brain tissue and cells were detected according to the requirements of the kit instructions.

### 2.13 Immunofluorescence

The expressions of p-PI3K, PI3K, AKT and p-AKT proteins in the hippocampus and SHSY-5Y cells were detected by IF. IF assays in tissues were performed in reference to the method of Wang et al. (Wang et al., 2016). The IF detection in SHSY-5Y cells was again performed according to the method of Marco et al. (Rosina et al., 2022).

### 2.14 Western blot detection

At the end of the drug intervention, cells were harvested, lysed by RIPA lysate and centrifuged to obtain total cell protein. The total protein concentration of cells was detected by BCA detection kit, and the protein concentration of cells in each group was adjusted according to the detection results. Subsequently, the protein loading buffer was added to desaturate the total cell protein by heating. Proteins were separated using SDS-PAGE gels, and the separated proteins were transferred to PVDF membranes. The PVDF membranes were blocked with 5% skim milk powder for 1 h at room temperature, followed by incubation with the corresponding primary antibodies at 4°C overnight. The next day, the primary antibody was recovered, and the secondary antibody was added to incubate the PVDF membrane for 1 h. Finally, the PVDF membrane was developed with ECL reagent.

### 2.15 Statistical

The significance of the differences between the different groups was analyzed with the one-way ANOVA analysis. The results are presented as mean ± SD, and the differences were considered significant at *p* < 0.05.

## 3 Results

### 3.1 Monomethyl lithospermate improves neurobehavioral deficit and reduces cerebral infarction caused by cerebral ischemia in middle cerebral artery occlusion rats

The recovery of neurobehavioral function after stroke is one of the important indicators for evaluating the therapeutic effect of drug treatment in clinical practice. In our study, we also evaluated the neurobehavior of rats in each group at 24 h after MACO. As shown in Figure 1B, the control rats did not undergo ischemic risk although they received the intervention of sham surgery, so the neurological functions of the rats were at a normal level similar with normal rats. The mice in the model group underwent MCAO showed obvious neurological deficit, and the score was significantly higher than that in the sham operation group. Interestingly, the neurologic deficit score of MOL pretreated rats was significantly lower than that of the model group at 24 h after ischemia-reperfusion (IR), suggesting MOL can improve the neurologic deficit of MCAO rats. To further evaluate the effect of MOL on MCAO rats, we observed the cerebral infarct area of the rats’ brain tissue by TTC staining. Results as shown in Figures 1C,D, the infarct volume of rats in the MACO group was significantly increased compared with that in the sham-operated group. In contrast, compared with the MCAO rats, the infarct volume of rats pretreated with MOL was significantly reduced.

### 3.2 Monomethyl lithospermate reduces neuronal damage caused by middle cerebral artery occlusion

**FIGURE 2:**
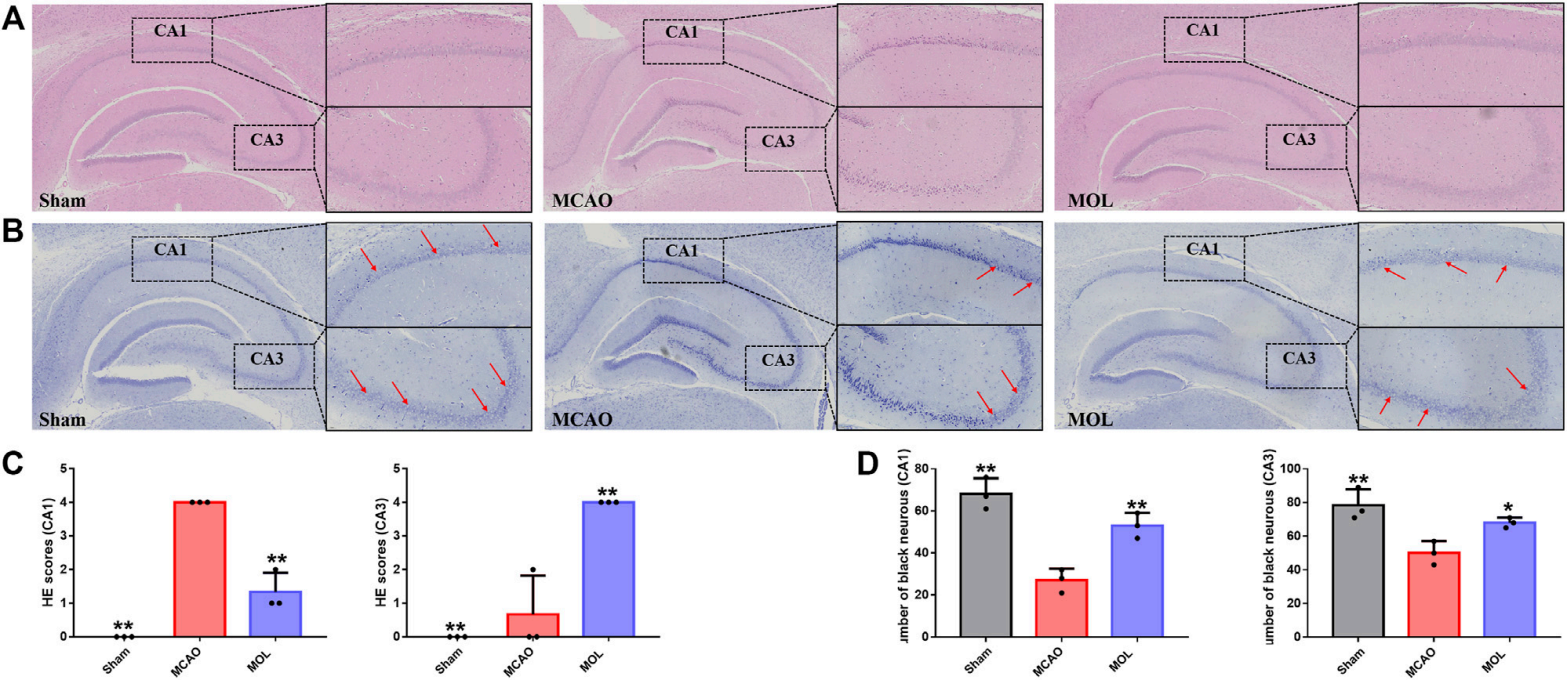
MOL can improve hippocampal neuronal cell damage in MCAO rats. **(A)** HE staining of rat hippocampus; **(B)** Nissl staining of rat hippocampus; **(C)** Pathological scores of HE staining in hippocampal CA1 and CA3 regions; **(D)** Pathological score of Nissl staining in the CA1, CA3 region of the hippocampus. Data expressed as Mean ± SD, **p < 0.01, *p < 0.05 vs. MCAO group.

The pathological changes of hippocampal tissue were analyzed by HE staining. The morphology and structure of hippocampal neurons in the sham operation group is normal, and the hippocampal pyramidal cells were closely arranged, with round nuclei, clear nucleoli, and abundant cytoplasm. However, after MCAO, the neuronal cells of rats were wrinkled and the volume was reduced, and the pyramidal cells of hippocampus were arranged in a reduced hierarchy and the structure was unclear. Interestingly, pre-treatment with MOL alleviated these pathological changes in the hippocampus of MCAO rats (Figures 2A,C). Similarly, the damage of neurons in hippocampal tissue of rats was observed by Nissl staining. Results as shown in Figures 2B,D, the morphology and structure of hippocampal nerve cells in normal rats were normal, and Nissl substance were abundant. However, MCAO rats showed a large number of hippocampal neuronal cell shrinkage and hyperchromatic pyramidal nuclei, accompanied by a significant decrease in the number of Nissl substance. Although similar conditions were observed in the MOL group, these pathological changes were significantly more optimistic. These results indicated that MOL intervention in advance could significantly alleviate the neuronal cell injury caused by MCAO in rats.

### 3.3 Monomethyl lithospermate inhibits oxidative stress and neuronal apoptosis in ischemic side of middle cerebral artery occlusion rats

**FIGURE 3:**
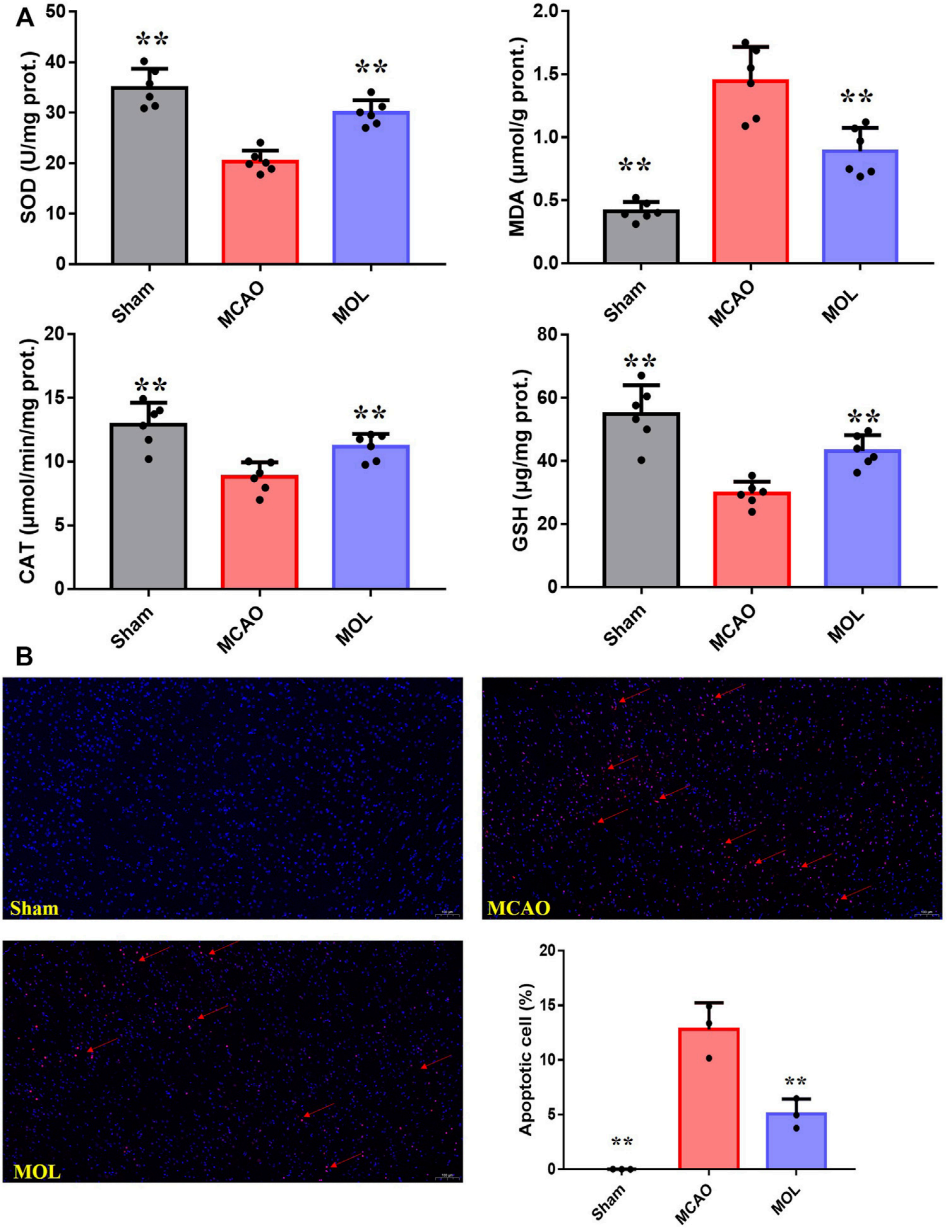
MOL can reduce the oxidative stress level and inhibit neuronal apoptosis in MCAO rats. **(A)** MOL up-regulated the levels of SOD, CAT and GSH in ischemic brain tissue of MCAO rats, and down-regulated the levels of MDA. **(B)** Tunel staining showed that MOL inhibited apoptosis of rat neuronal cells induced by MCAO treatment. Data expressed as Mean ± SD, **p < 0.01, *p < 0.05 vs. MCAO group.

Oxidative stress and neuronal apoptosis are considered to be the main mechanisms of brain injury after cerebral IR. In our study, we examined the level of oxidative stress in the ischemic part of the brain tissue of MCAO rats. As shown in Figure 3A, SOD and CAT activities were significantly decreased, GSH levels were also significantly decreased, and MDA levels were significantly increased in the MCAO group compared with the sham group. And MOL recovered the activities of SOD and CAT, increased the level of GSH, and decreased the content of MDA. These results suggest that MOL can prevent the increase of oxidative stress level in brain tissue of rats after MCAO. In addition, strong red fluorescence was observed in the nucleus of MCAO group in TUNEL staining assay (Figure 3B), which indicated severe DNA fragmentation and apoptosis of neurons. Interestingly, pretreatment with MOL attenuated DNA damage and apoptosis induced by MCAO.

### 3.4 Monomethyl lithospermate alleviates cell damage, mitochondrial membrane potential loss and apoptosis of SHSY-5Y cells after oxygen and glucose deprivation/reoxygenation

**FIGURE 4:**
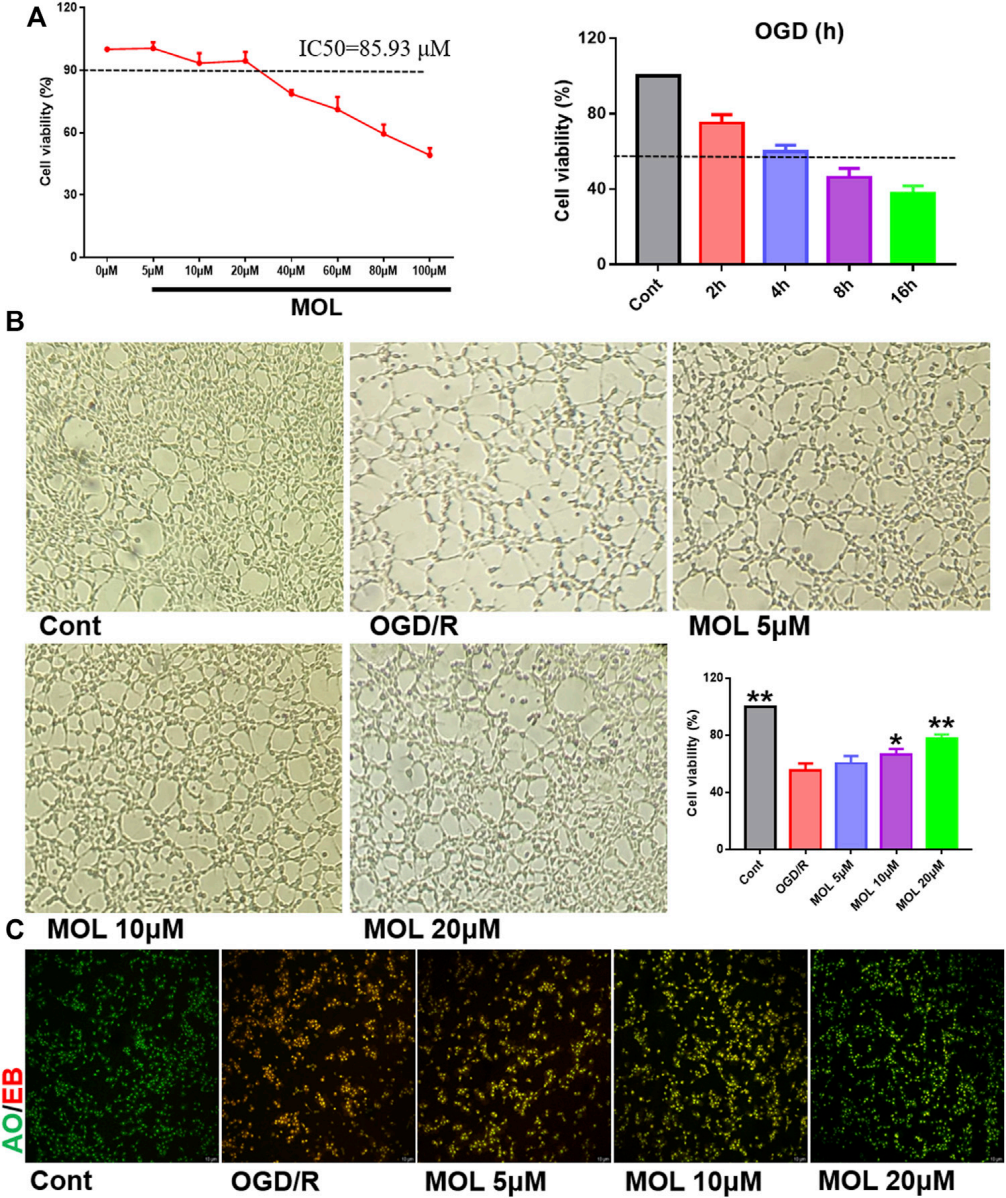
MOL protected SHSY-5Y cells from damage caused by OGD/R treatment. **(A)** Effects of different concentrations of MOL (0–100 μM) on the viability of SHSY-5Y cells, and the effect of different OGD times on cell viability; **(B)** Effects of different concentrations of MOL (5, 10, 20 μM) on morphology and viability of SHSY-5Y cells treated with OGD/R. **(C)** AO-EB staining of SHSY-5Y cells treated with different concentrations of MOL (5, 10, 20 μM) and OGD/R. Data expressed as Mean ± SD, **p < 0.01, *p < 0.05 vs. OGD/R group.

**FIGURE 5:**
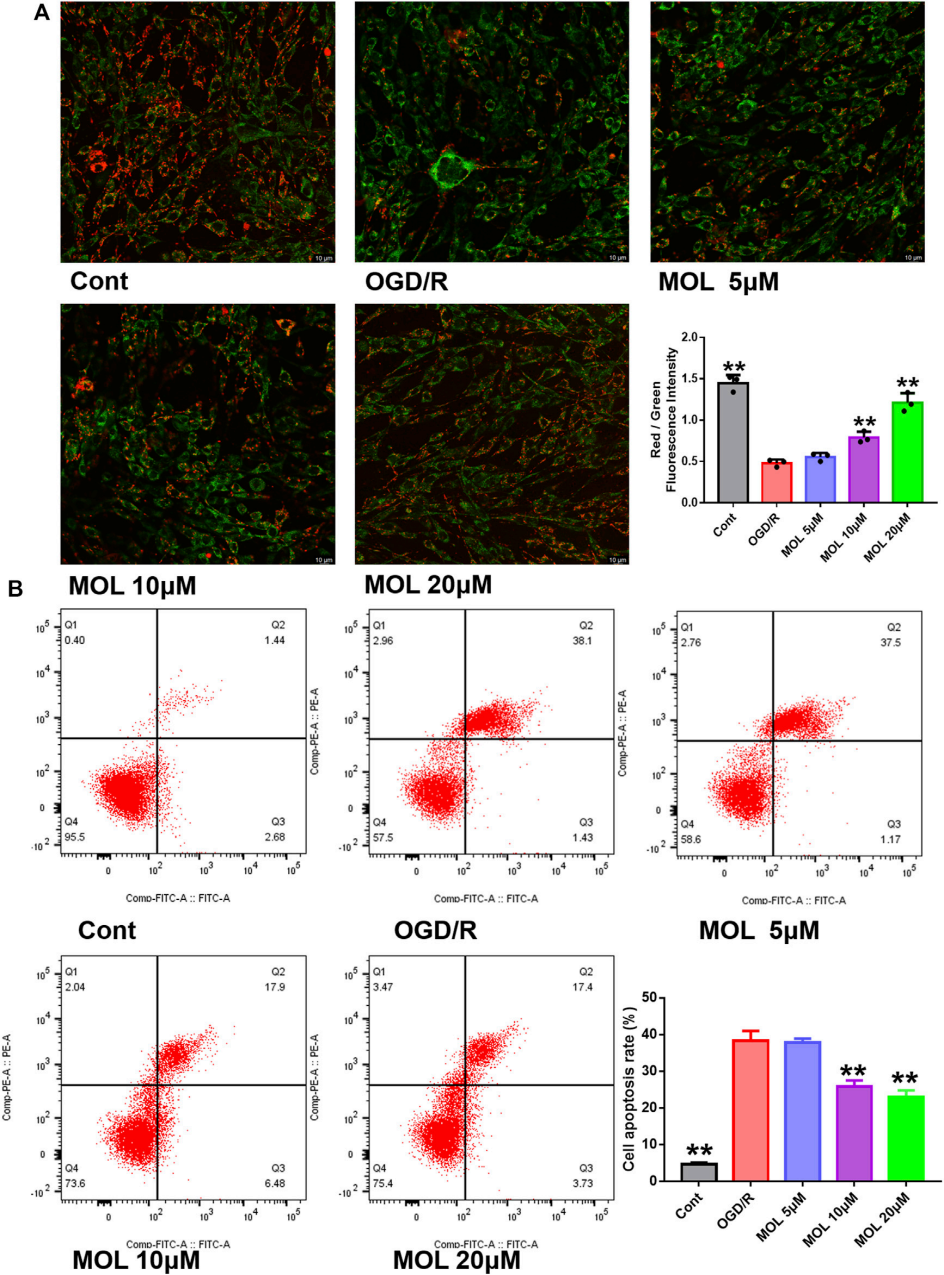
MOL inhibited the MMOP collapse and apoptosis of SHSY-5Y cells induced by OGD/R treatment. **(A)** JC-1 staining was used to observe the changes of MMOP in SHSY-5Y cells treated with MOL (5, 10, 20 μM) and OGD/R. **(B)** Flow cytometry was used to detect the apoptosis of SHSY-5Y cells after MOL (5, 10, 20 μM) and OGD/R treatment. Data expressed as Mean ± SD, **p < 0.01, *p < 0.05 vs. OGD/R group.

The OGD/R-treated SHSY-5Y cell model was constructed *in vitro* to study the neuroprotective effect of MOL. Before the start of the experiment, we screened the working concentration of MOL by CCK8 assay, which showed no cytotoxicity to SHSY-5Y cells at MOL concentration less than or equal to 20 μM (Figure 4A). We also investigated the effect of OGD deprivation time on cell viability, and finally selected 4 h OGD deprivation time as the OGD/R model (Figure 4A). Subsequently, it was also observed that OGD/R treatment significantly reduced the viability of SHSY-5Y cells, while MOL intervention could restore the viability of SHSY-5Y cells (Figure 4B). Similarly, we also observed that OGD/R treatment would destroy the integrity of cell membrane by AO-EB staining, suggesting that SHSY-5Y cells showed obvious cell damage after OGD/R. Interestingly, MOL intervention can alleviate the damage of SHSY-5Y cells induced by OGD/R (Figure 4C). Figure 5A shows similar results, the MMOP of SHSY-5Y cells was significantly reduced after OGD/R treatment, but MOL treatment restored the MMOP of SHSY-5Y cells. In addition, the apoptosis rate of SHSY-5Y cells was detected by flow cytometry, and the results were shown in Figure 5B. OGD/R treatment could significantly induce cell apoptosis, while MOL treatment could inhibit the apoptosis of SHSY-5Y cells caused by OGD/R.

### 3.5 Monomethyl lithospermate reduces ROS and intracellular oxidative stress in SHSY-5Y cells

**FIGURE 6:**
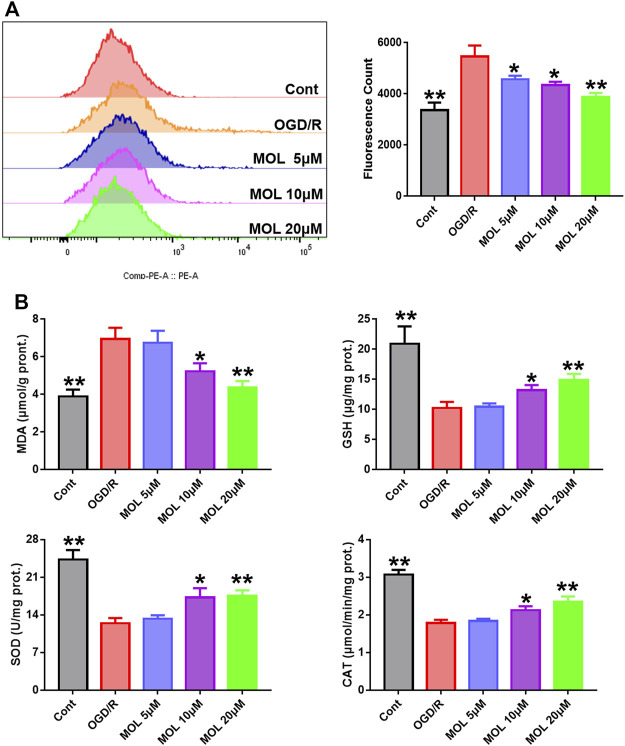
MOL intervention can improve the oxidative stress level of SHSY-5Y cells treated with OGD/R. **(A)**: MOL (5, 10, 20 μM) inhibited ROS production in SHSY-5Y cells treated with OGD/R; **(B)** MOL (5, 10, 20 μM) increased the levels of SOD, CAT and GSH in SHSY-5Y cells treated with OGD/R, and decreased the levels of MDA. Data expressed as Mean ± SD, **p < 0.01, *p < 0.05 vs. OGD/R group.

*In vivo* experiments, we observed that MOL treatment could significantly improve the oxidative stress state of rat brain tissue. Therefore, we also examined intracellular oxidative stress *in vitro*. As shown in Figure 6A, intracellular ROS levels were significantly increased in cells receiving OGD/R treatment compared with the control group. Interestingly, treatment with different concentrations of MOL significantly inhibited ROS production in SHSY-5Y cells. Similarly, we also found that OGD/R treatment significantly increased the level of MDA, inhibited the activities of SOD and CAT, and decreased the level of GSH. However, MOL treatment could reverse the oxidative stress induced by OGD/R treatment (Figure 6B).

### 3.6 Monomethyl lithospermate activated PI3K/AKT of middle cerebral artery occlusion rats and SHSY-5Y cells

**FIGURE 7:**
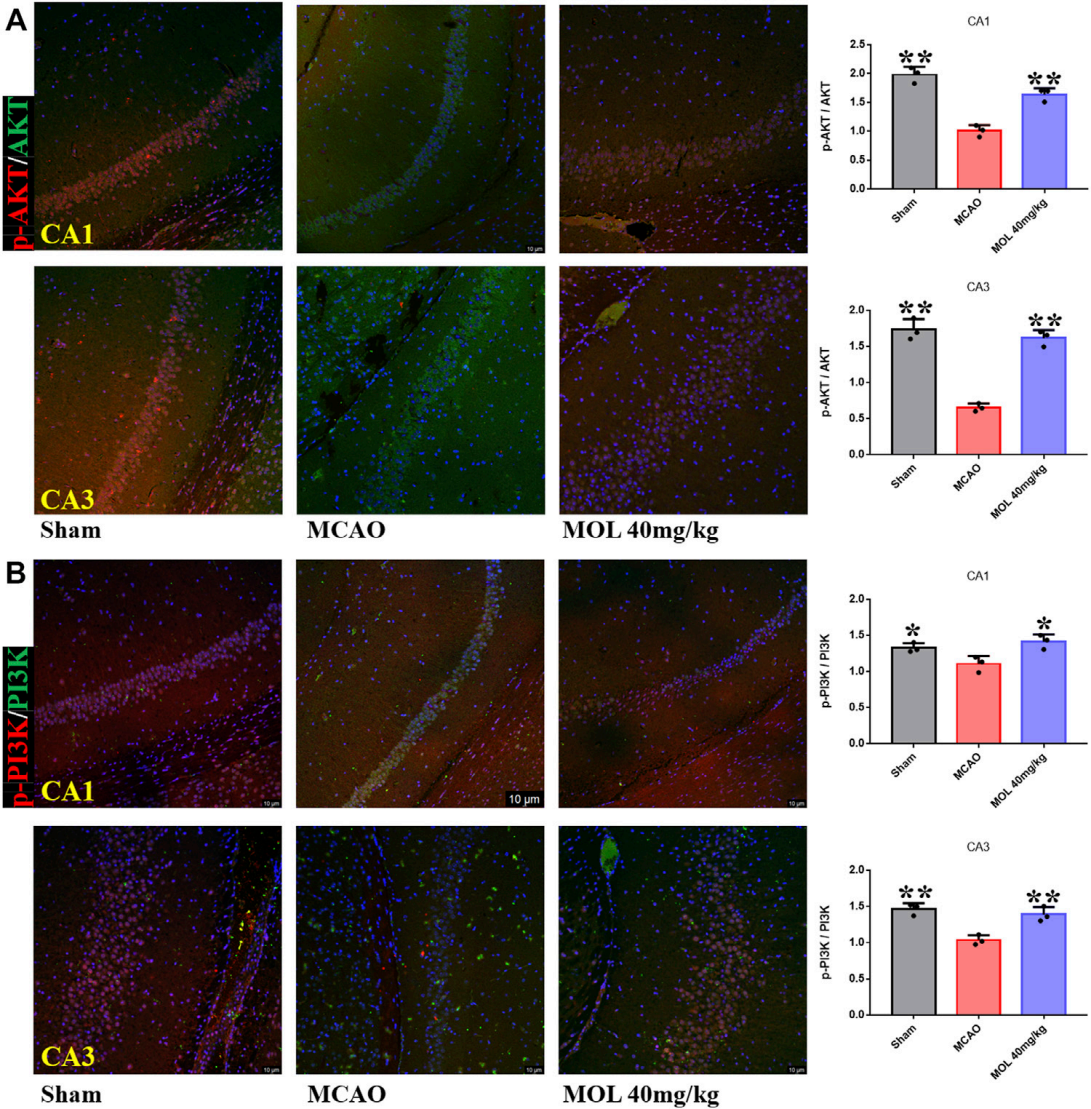
MOL treatment can activate PI3K/AKT pathway in hippocampus of MCAO rats. **(A)** MOL (72.4 μM/kg) treatment up-regulated p-Akt/AKT in hippocampal CA1 and CA3 regions of MCAO rats; **(B)** MOL (72.4 μM/kg) treatment up-regulated p-PI3K/PI3K in hippocampal CA1 and CA3 regions of MCAO rats. Data expressed as Mean ± SD, **p < 0.01, *p < 0.05 vs. OGD/R group.

**FIGURE 8:**
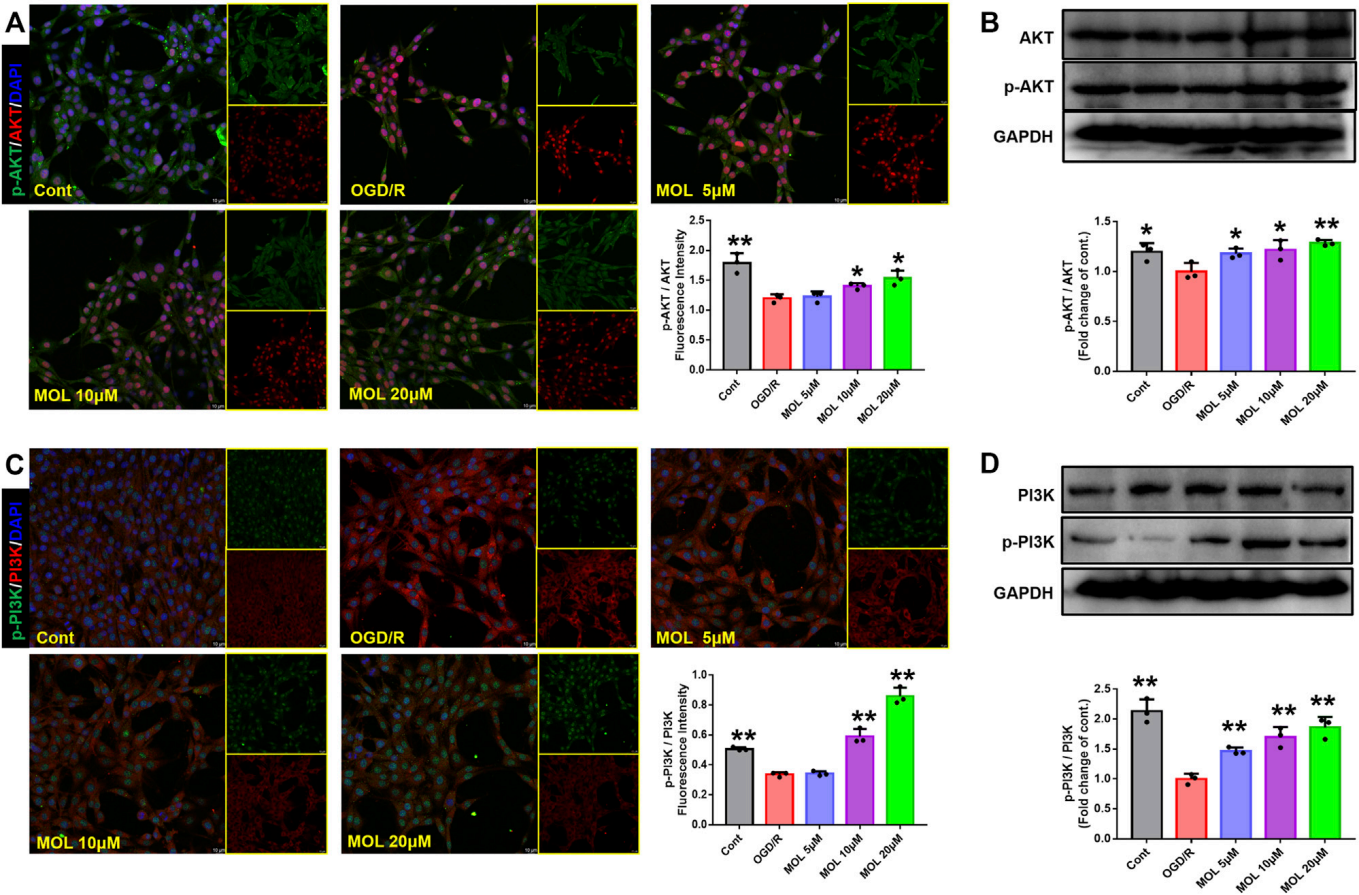
MOL treatment can activate the PI3K/AKT pathway in SHSY-5Y cells induced by OGD/R. **(A)** and **(B)** MOL (5, 10, 20 μM) treatment can up-regulate the phosphorylation level of AKT and increase the p-Akt/AKT ratio in SHSY-5Y cells induced by OGD/R. **(C)** and **(D)** MOL (5, 10, 20 μM) treatment can up-regulate the level of PI3K phosphorylation in SHSY-5Y cells induced by OGD/R, and increase the ratio of p-PI3K to PI3K. Data expressed as Mean ± SD, **p < 0.01, *p < 0.05 vs. OGD/R group.

The PI3K/AKT pathway plays an important role in cellular oxidative stress and apoptosis. In our study, effects of the MOL on PI3K/AKT pathway were also observed in MCAO rats and OGD/R-treated SHSY-5Y cells. IF staining showed that p-Akt/AKT and p-PI3K/PI3K in the hippocampus of rats after MCAO treatment were significantly lower than those in the sham operation group, suggesting that MCAO treatment could inhibit the phosphorylation of AKT and PI3K in hippocampal neurons. However, pretreatment with MOL alleviated these changes and increased the phosphorylation of AKT and PI3K (Figure 7). Similar results were observed in cell experiments. Through IF and WB experiments, it was found that p-Akt/AKT and p-PI3K/PI3K were significantly decreased after OGD/R treatment, but MOL treatment could significantly increase the ratio of p-AKT to p-PI3K. Taken together, our study confirmed that MOL intervention significantly activated the PI3K/AKT pathway in MCAO rats and OGD/R-treated SHSY-5Y cells (Figure 8).

### 3.7 Monomethyl lithospermate ameliorates ischemia-reperfusion-induced nerve damage by activating PI3K/AKT

**FIGURE 9:**
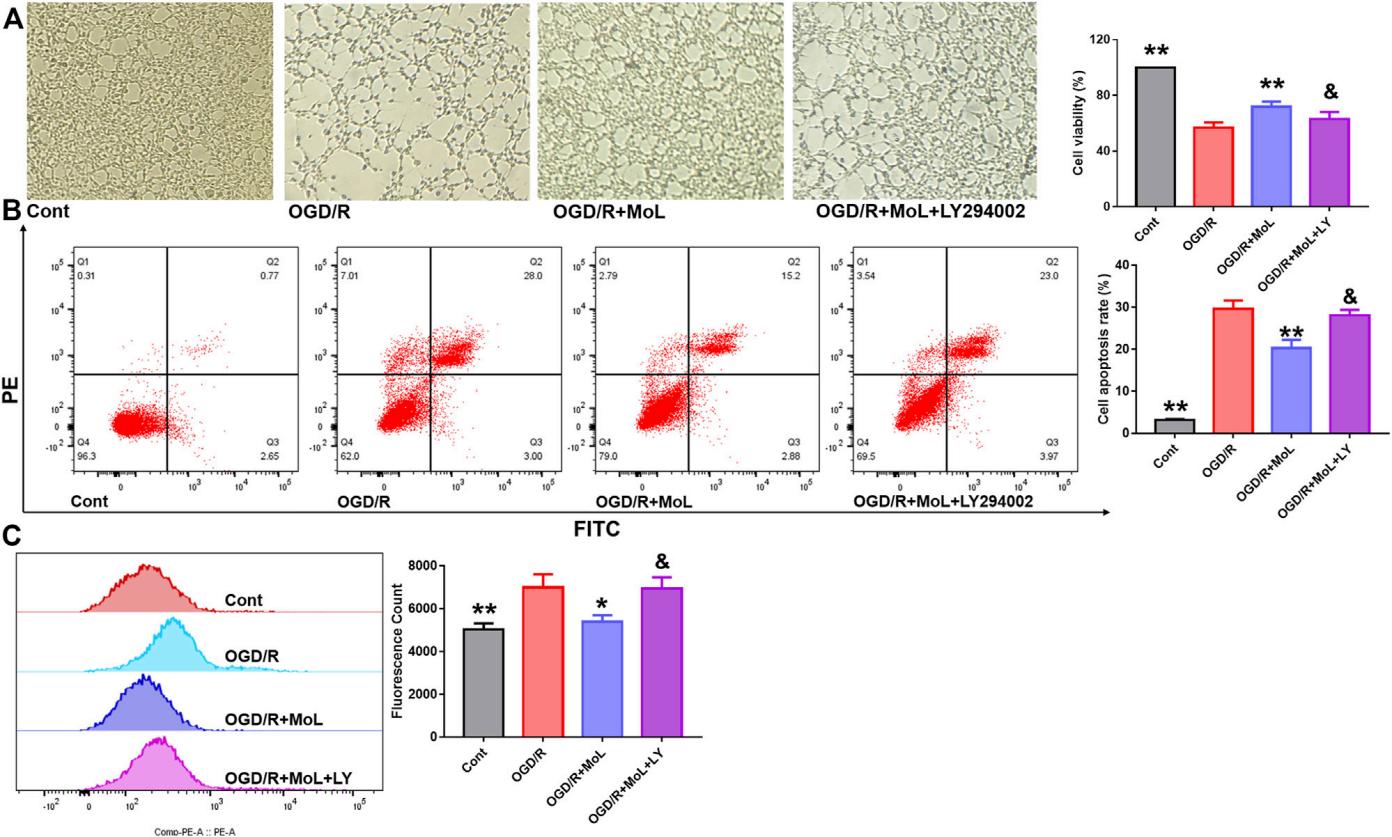
MOL can partially inhibit OGD/R-induced apoptosis and oxidative stress in SHSY-5Y cells by activating PI3K/AKT pathway. **(A)** The PI3K/AKT pathway inhibitor LY294002 (10 μM) prevented MOL (20 μM) from saving the viability of OGD/R-treated SHSY-5Y cells; **(B)** LY294002 (10 μM) inhibited the anti-apoptotic effect of MOL (20 μM) on OGD/R-treated SHSY-5Y cells; **(C)** LY294002 (10 μM) inhibited the anti-ROS effect of MOL (20 μM) on OGD/R-treated SHSY-5Y cells. Data expressed as Mean ± SD, **p < 0.01, *p < 0.05 vs. OGD/R group.

To further investigate whether the neuroprotective effect of MOL was related to the PI3K/AKT pathway. LY294002, an inhibitor of the PI3K/AKT pathway, was used to observe the protective effect of MOL on SHSY-5Y. Results as shown in Figure 9, MOL intervention significantly rescued OGD/R-induced damage and apoptosis in SHSY-5Y cells and reduced the level of intracellular ROS. Interestingly, the protective effect of MOL on SHSY-5Y was greatly compromised in the presence of LY294002. These results suggest that the neuroprotective effect of MOL is related, at least in part, to activation of the PI3K/AKT pathway.

## 4 Discussion

Recently, increasing literatures have reported lots of extracts and compounds from herbal medicines could be used to treat various brain diseases *via* protecting neurons in brain from injury induced by various factors (Zhu et al., 2020; Li et al., 2022; Lyu et al., 2022; Peng et al., 2022; Zhong et al., 2022). To the best of our knowledge, our present study is the first evidence that monomethyl lithospermate (MOL), which is an active compound reported from the *Salvia miltiorrhiza* Bge., has potential improving effects against ischemic stroke injury, and studied its involved mechanism of actions.

It is suggested that oxidative stress and its related molecular events plays important roles in the development of stroke (Chamorro et al., 2016; Zhu et al., 2021). The cerebral ischemia and ischemia reperfusion (I/R) after stroke would result in a series of cell responses, over-production of reactive oxygen species (ROS) and reduction of antioxidant enzymes, and subsequently lead to oxidative stress (Xuan et al., 2019; Zhu et al., 2021). The over-produced ROS can not only result in lipid peroxidation and oxidation of protein and DNA, but also lead to cell apoptosis *via* activation of multiple cell signaling pathways. Consequently, previous studies have indicated that suppression of oxidative stress might be a promising feasible therapeutic way for treating stroke (Li et al., 2021). In the present study, a stroke animal model was constructed by using the middle cerebral artery occlusion (MCAO) method in rats. From the results of our present study, it is suggested that MOL has promising therapeutic effects against stroke with improvement of the brain infarct volume, neurological deficit score and cells apoptosis of brain. Next, we further determined the anti-apoptotic effect of MOL on the oxygen glucose deprivation/reoxygenation (OGD/R) stimulated SHSY-5Y cells. We found that MOL can increased the cell viability and mitochondrial membrane potential (MMOP) of OGD/R induced SHSY-5Y cells, whereas reduced the apoptosis ratio of OGD/R induced SHSY-5Y cells. All these results mentioned above suggested that MOL can inhibit the I/R induced damage in neuron cell *via* suppression of cell apoptosis.

It is reported that the excessive ROS could result in direct oxidative damage of molecules to result in cell dysfunction and apoptosis (Li et al., 2020). So, how to reduce the ROS levels in brain after stroke is important for protecting the function of neuron cells, which would be helpful for the recovery post stroke. In our present results, it is indicated that MOL could significantly reduce the ROS levels in OGD/R induced SHSY-5Y cells. Nowadays, it is reported that PI3K/Akt signaling plays predominant effects for scavenging the over-produced ROS in cells (Liu et al., 2018; Zhang et al., 2022). The activated PI3K/Akt signaling would result in intranuclear accumulating of the nuclear factor erythroid 2-related factor 2 (Nrf2) protein which is a nuclear transcription factor, and the Nrf2 protein is important for the production of antioxidant enzymes, such as SOD, CAT, GSH-Px, *etc*., leading to the scavenging of ROS in cells (Zhang Q. et al., 2021; Zhu et al., 2021). Furthermore, it is also demonstrated that activated PI3K/Akt signaling can suppress the pro-apoptotic molecules to further block the apoptotic signaling and protect the cells from apoptosis (Zhang S. Q. et al., 2021). Interestingly, the obtained results from the present study revealed MOL could up-regulate the PI3K/Akt signaling in the OGD/R induced SHSY-5Y cells. All these results mentioned above suggested MOL can suppress the apoptosis in SHSY-5Y cells induced by OGD/R *via* activation of PI3K/Akt signaling. Of course, our current study also has certain limitations. In the *in vivo* experiment, we only examined the effect of a single dose of MOL, and such an evaluation is far from sufficient to elucidate the neuroprotective mechanism of MOL. However, we are conducting further experiments to explore the neuroprotective effect of MOL, including the dose and time effect of MOL, and the influence of the participation of related inhibitors on the efficacy of MOL, in order to clarify the mechanism of action of MOL in improving IS.

In summary, our present experimental results *in vivo* and *in vitro* demonstrated that MOL has potential protective effects on neuron cells from damage induced by IR. Furthermore, the possible mechanisms actions are closely related to suppression of cell apoptosis through activating PI3K/Akt signaling pathway.

## Data Availability

The original contributions presented in the study are included in the article/supplementary materials, further inquiries can be directed to the corresponding authors.
